# Association between HLA-E gene polymorphism and unexplained recurrent spontaneous abortion (RSA) in Iranian women

**Published:** 2016-07

**Authors:** Maryam Fotoohi, Nasrin Ghasemi, Seyed Ali Mirghanizadeh, Mahmood Vakili, Morteza Samadi

**Affiliations:** 1 *Reproductive Immunology Research Center, Shahid Sadoughi University of Medical Sciences, Yazd, Iran.*; 2 *Recurrent Abortion Research Center, Research and Clinical Center for Infertility, Shahid Sadoughi University of Medical Sciences, Yazd, Iran. *; 3 *Immunology Department, Faculty of Medicine, Shahid Sadoughi University of Medical Sciences, Yazd, Iran. *; 4 *Community and Preventive Medicine Department, School of Medicine, Shahid Sadoughi University of Medical Sciences, Yazd, Iran. *; 5 *Research Center for Food Hygiene and Safety, Shahid Sadoughi University of Medical Sciences, Yazd, Iran.*

**Keywords:** *Spontaneous Abortions*, *HLA-E antigen*, *Polymorphism*

## Abstract

**Background:**

Human leukocyte antigen-E (HLA-E)is a non-classical major histocompatibility complex (MHC) class I antigens which expressed on extra villous cytotrophoblast, which interacts with NKG2A, is an inhibitory receptor on natural killer (NK) cells and leading to down regulation of immune response in the maternal-fetal interface and provides maternal immune tolerance of the fetus.

**Objective:**

This study was designated to investigate the gene frequencies of E0101 and E0103 in HLA-E gene in Iranian women with recurrent spontaneous abortion (RSA).

**Materials and Methods:**

Amplification Refractory Mutation System (ARMS-PCR) technique was carried out to detect polymorphism in exon 3 of the HLA-E gene in women with RSA and controls (n=200). Differences between groups were analyzed by SPSS19 software using ^2^ test.

**Results:**

There was no significant difference in the allele frequencies of the ***HLA-E polymorphism between RSA and fertile controls ***but HLA-E 0101/0103 heterozygous genotype was found to be significantly higher in RSA group (p=0.006, OR=1.73), so this genotype might confer susceptibility to RSA.

**Conclusion:**

Our results suggest that HLA-E 0101/0103 heterozygous genotype leads to increase of RSA risk. It seems that by genotyping of HLA-E polymorphism, we can predict the risk of RSA in infertile women.

## Introduction

Recurrent spontaneous abortion (RSA), is a condition defined as three or more miscarriage prior to the 24^th^ week of gestation (1). This affects 1-2% of women who are pregnant (2, 3). RSA causes are commonly include chromosomal abnormalities, anti-phospholipid syndrome, acquired and inherited thrombophilia, autoimmune diseases, uterine pathologies, environmental factors, maternal infections, endocrine dysfunctions, and in nearly 50% of RSA cases unknown factors (idiopathic) are cause of this complication (4-8). In fact, a significant proportion of idiopathic RSA may be due to immune factors (2). 

During pregnancy, despite of the fetus being regarded as a semi-allograft to the mother, fetus is protected from rejection by maternal immune response. However, in normal pregnancy several tolerance mechanisms have been demonstrated to circumscribes the maternal immune response (3, 9, 10). Among these, the expression of human leukocyte antigen-E (HLA-E) by cytotrophoblasts has been shown to play an important role in creating a tolerogenic condition at the feto-maternal interface. HLA-E is a non-classical HLA class Ib molecule which has a wide tissue distribution. HLA-E interacts with CD94/NK G2A complex, a lectin type NK cell inhibitory receptor, plays a fundamental role in the inhibition of NK cell activity (11, 12). Uterine natural killer cells (UNK cells) are resident in the endometrium and constitute 70% of endometrial leucocytes and are adjacent to fetal trophoblast cells in maternal-fetal interface, that these cells constitute the most predominant leucocyte population during implantation time and early pregnancy (13). HLA-E has two non-synonymous alleles (HLA-E0103 and HLA-E0101). HLA-E0101 allele differs from HLA-E0103 by an amino acid substitution (arginine to glycine at position 107 of the a2 heavy-chain domain) and also both of them have a difference cell surface expression (14). 

NK cell inhibitory or activating functions are controlled by interactions of HLA-E; so it shows the urgency HLA-E variant allele's investigation which may affect the development of RSA. This has motivated us to investigate the HLA-E polymorphism in normal fertile women and RSA in order to discover a possible correlation between the HLA-E polymorphisms and RSA.

## Materials and methods

**Patients**

In this analytic cross-sectional study, peripheral blood samples were obtained from 200 Iranian women who referred to the infertility center, Yazd, Iran between December 2013 to December 2014. Consent was obtained from all subjects who are participated in this study, and the Institutional Ethics Committee of Shahid Sadoughi University of Medical Sciences approved protocols of this study. 

Case group had three or more RSA which no apparent conventional causes of abortion such as anatomical, chromosomal, hormonal, thrombophilia and women in control group were without any history of abortion with at least one healthy child. Following data were obtained from all subjects: number of spontaneous abortions, age, and time of spontaneous abortion during pregnancy, age at each spontaneous abortion, and occurrence of bleeding and pain during the event.

**DNA Isolation**

Blood samples of both groups were collected in tubes containing EDTA. DNA was extracted from the peripheral blood sample using routine salting out procedure (15).

**ARMS-PCR analysis**

The E0101 and 0103 variants at codon 107 of HLA-E were identified by primer Amplification Refractory Mutation System (ARMS-PCR). A method which was originally designed for the detection of known sequence by using just two pairs of primers in a single PCR tube, this method can simultaneously amplify both mutant and wild type alleles, plus it allows for the amplification of an internal DNA control: two primers correspond to the polymorphic site and are complementary (in opposite directions) with the 3´-terminal nucleotides. 

**Figure 1:**
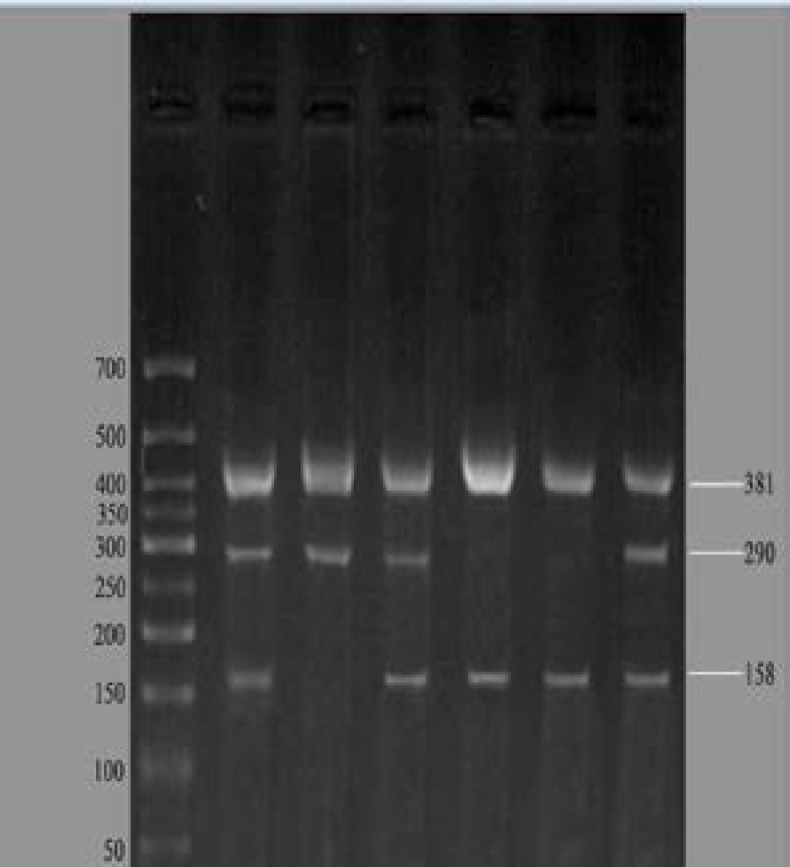
ARMS-PCR for exon 3 codon 107. The band control was of 381 bp, whereas at polymorphic site, adenine and guanine produced band of 290 and 158 bp.

The other two primers are external to the first set and target to non-polymorphic sites. The primers and the expected product sizes are shown in table I. The amplification reaction was performed in 26 μl volumes. One pair the PCR mixture contained 2 μl of DNA, 12 μl master mix, 8 μl H_2_O and 1 μl of each four primers. Reaction conditions were carried out in thermo cycler (ABI, USA), 94^o^C for 5 min, 35 cycles at 94^o^C for 1 min, 67^o^C for 1 min, and 72^o^C for 1 min followed by an extension at 72^o^C for 10 min. The digestion product were subjected to 2% agarose gel electrophoresis (paya pajohesh Eps-7601-Iran) and stained with Green viewer (containing ethidium bromide), and then visualized under UV light. The product sizes at ARMS-PCR codon 107 are shown in figure 1. 

**Statistical analysis**

Genotypes frequencies in RSA and fertile control groups were compared with Hardy-Weinberg expectations using χ^2^ analysis. The data were processed by SPSS 19 software (Statistical Package for the Social Sciences, Inc., Chicago, IL, USA). Odds ratios were calculated with a confidence interval of 95% and p<0.05 was considered significant.

## Results

In this study, polymorphism at codon 107 of HLA-E exon 3 was detected through ARMS-PCR. The control was a band of 381 bp, whereas at polymorphic site, adenine and guanine produced band of 290 and 158 bp, respectively (Figure 1). Table II shows the frequency of HLA-E 0101 and 0103 alleles in the RSA and fertile controls. Our results show that the frequency of HLA-E 0101 allele is higher than HLA-E0103 allele in RSA (Chi=1.45, OR=1.19). We found no significant difference in frequency of HLA-E alleles in RSA when compared with that in the controls (p=0.229).

The distribution of HLA-E genotypes is shown in table III. Our results show that, the frequency of HLA-E 0101/0103 heterozygote genotype is higher in RSA than in normal fertile control (chi=4.37, p=0.006, OR=1.73, 95% CI=1.14-2.63). So this polymorphism HLA-E 0101/0103 heterozygous is associated with susceptibility to RSA. The difference in the frequency of HLA-E 0101/0103 heterozygous genotype in RSA group was statistically significant when compared to the controls. However, the frequency of the HLA-E 0101/0101 homozygote was lower in RSA group than in fertile controls. The HLA-E 0103 homozygotes showed lower frequency in RSA group than in controls (p=0.013, OR=0.56, 95% CI=0.35-0.91). The analysis of results indicated that the HLA-E genotype frequencies were in agreement with a Hardy-Weinberg equilibrium in this study.

**Table I:** Primers used for the determination of HLA-E exons 3 (codons 107) polymorphisms by ARMS analysis

**Polymorphism**	**Primer sequence**	**Product size (bp)**
Codon 107 (A→G)	5´-CAAAATGCCCACAGGGTGGTGGCGACGGG-3´	Control: 381 A: 290 G: 158
	5´-ATGCATGGCTGCGAGCTGGGGCCCGAAA-3´	
	5´-GAACTGTTCATACCCGCGGAGGAAGCGACC-3´	
	5´-GGAGATGGGAGAGTAGCCCTGTGGACCCTC3´	

**Table II:** Frequency of HLA-E alleles in RSA and fertile female controls (n=200)

**HLA-E Alleles**	**RSA**	**Control**	**OR**	**%95CI**	**p-value**
0101 (A)	213 (53)	196 (49)	1.19	0.85-1.58	0.229
0103 (G)	187 (47)	103 (51)	0.84	0.63-1.12	0.229

Data presented as n (%). Evaluated by Chi-square test and p≤0.05 was considered significant.

HLA-E: human leucocyte antigen E

OR: odds ratio

95% CI: 95% confidence interval.

**Table III:** Frequency of HLA-E genotypes in RSA and fertile female controls (n=200)

**HLA-E genotypes**	**Control**	**RSA**	**OR** *	**%95CI**	**p-value**
0101/0101	60 (30)	55 (27.5)	0.89	0.56-1	0.58
0103/0103	64 (32)	42 (21)	0.56	0.35-0.91	0.013
0101/0103	76 (38)	103 (51.5)	1.73	1.14-2.63	0.006

Data presented as n (%). Evaluated by Chi-square test and p≤0.05 was considered significant.

HLA-E: human leucocyte antigen E

OR: odds ratio

95% CI: 95% confidence interval.

* OR was computed for each genotype in considering the existence of other genotypes for example 0101/0101 contrast with 0103/0103 or 0101/0103.

## Discussion

In this study, we hypothesized that HLA-E polymorphisms might cause susceptibility to RSA. By comparing the HLA-E0101 and HLA-E0103 polymorphisms of HLA-E gene between RSA females and fertile female controls, higher frequencies of HLA-E0101 were observed in RSA women. However, we found no significant difference for these alleles in recurrent abortion. Expression of HLA-E at feto-maternal interface is implicated in successful pregnancy because it is capable to down regulate maternal immune response (14). 

HLA-E antigens are believed to help to fetal survival through interaction with CD94/NKG2A complex, a NK inhibitory receptor, and inhibition of NK cells (12). HLA-E is a non-classical MHC class I genes (class Ib), which is located on chromosome 6p21.3, between the classical HLA class Ia genes HLA-A and HLA-C (16). This gene is characterized by a limited polymorphism and low cell surface expression (17). The nonamers peptide leader derived from classical class I antigens including HLA-C, -A, -B, and preferentially HLA-G are necessary for surface expression of HLA-E on the surface cell (18, 19). HLA-E is expressed in B cells, T cells, activated T lymphocytes, and cells such as placenta and trophoblast cells (16).

Trophoblast cells are devoid of classical HLA class I molecules, providing a protection against cytotoxic T lymphocyte (CTL). In addition, the presence of non-classical, HLA-E and HLA-G at feto-maternal interface helps to down regulation and inhibition of NK cells (20, 21). HLA-E at feto-maternal interface are associated with down regulation of NK cells activity (11). There are two major allele of HLA-E, E0101 and E0103 (18). There are two alleles of HLA-E, which substitution of an arginine with a glycine at position 107 creates them, glycine (E0103; HLA-E^G^) or arginine (E 0101; HLA-E^R^) (22-24). Each of them have a different levels of cell surface expression of HLA-E molecules which might be caused by their different peptide affinity and thermal stability (17). Biological functions and surface expression of two non-synonymous alleles of HLA-E are different. In this case, E0101 always has a lower surface expression compared to HLA-E0103 (16, 25). Analysis of empty HLA-E and its complex with peptide revealed that the HLA-E0103 allele was more thermally stable than the HLA-E0101 allele (18, 26). 

A study by Tripathi *et al* showed that lower stability and expression of HLA-E 0101 may leading to ineffective inhibition of uNK cells and hence may be associated with RSA (16). From this it can be conclude that the HLA-E0103 induces uNK and ultimately prevents rejection of the fetus. Our results show HLA-E 0103 is higher in controls (38%) than patients (21%). The results of Mosaad *et al* work revealed that higher number of HLA-E0101 alleles in Egypt population and that the HLA-E allele frequencies in this population were similar to those reported in various populations such as Indians, African American, Caucasian and Hispanic populations Africans and Gaza strip-Palestine also Indo-Asian, Afro-Caribbean, and Euro-Caucasoid (2, 16, 27-29). 

On the other wise , the gene frequency of E0103 is significantly higher than E0101 in the Japanese and Chinese populations (27, 30). In addition, Many reports have been shown that there is no significant difference in the frequency of HLA-E alleles between RSA females and normal controls (2, 31, 32). In line with this studies, our study revealed that there was no significant association in HLA-E alleles (E0101, E0103) between RSA females and normal subjects (p=0.229). The difference in the distribution of HLA-E alleles between different studies may be due to geothnic variation (14).

There are three possible genotypes of HLA-E alleles -HLA-E 0101/0101, HLA-E 0103/0103 and HLA-E 0101/0103 (33). Several studies have shown that association between HLA-E polymorphisms and outcomes in various situations and diseases (34). Our results revealed that, HLA-E 0103/0103 homozygous that is higher in controls is associated with maintaining the fetus in a successful pregnancy by inhibition of NK. A study by Lajoie *et al* showed that the protective effect of HLA-E0103/0103 genotype on HIV-1 infection in Zimbabwean women (24). 

## Conclusion

Conclusively, our results suggest a protective function of HLA-E 0103/0103 genotype which is higher in RSA. It provides better inhibition of NK cells and allows pregnancy maintenance in fertile controls and though HLA-E 0101 is related to ineffective inhibition of NK. Our data showed that the HLA-E0101/0103 heterozygotes are related to fetus survival.

## References

[1] Dawood F, Quenby S, Farquharson R (2003). Recurrent miscarriage: an overview. Rev Gynaecol Pract.

[2] Matter TF, Sharif FA (2013). HLA-G and HLA-E Gene polymorphisms in idiopathic recurrent spontaneous abortion women in Gaza strip-Palestine. Int J Reprod Contracept Obstet Gynecol.

[3] Arjmand F, Samadi M (2015). Association of 14-bp insertion/deletion polymorphism of HLA-G gene with idiopathic recurrent miscarriages in infertility center patients in Yazd, Iran. J immunotoxicol.

[4] Toth B, Jeschke U, Rogenhofer N, Scholz C, Würfel W, Thaler CJ (2010). Recurrent miscarriage: current concepts in diagnosis and treatment. J Reprod Immunol.

[5] Lee SK, Kim JY, Lee M, Gilman‐Sachs A, Kwak‐Kim J (2012). Th17 and regulatory T cells in women with recurrent pregnancy loss. Am J Reprod Immunol.

[6] Matthiesen L, Kalkunte S, Sharma S (2012). Multiple pregnancy failures: an immunological paradigm. Am J Reprod Immunol.

[7] Ostojić S, Pereza N, Volk M, Kapović M, Peterlin B (2008). Genetic Predisposition to Idiopathic Recurrent Spontaneous Abortion: Contribution of Genetic Variations in IGF‐2 and H19 Imprinted Genes. Am J Reprod Immunol.

[8] Abdollahi E, Tavasolian F, Ghasemi N, Mirghanizadeh SA, Azizi M, Ghoryani M (2015). Association between lower frequency of R381Q variant (rs11209026) in IL-23 receptor gene and increased risk of recurrent spontaneous abortion (RSA). J Immunotoxicol.

[9] Arjmand F, Ghasemi N, Mirghanizadeh SA, Samadi M (2016). The balance of the immune system between HLA-G and NK cells in unexplained recurrent spontaneous abortion and polymorphisms analysis. Immunol Res.

[10] Tavasolian F, Abdollahi E, Samadi M (2014). Association of the IL4R single-nucleotide polymorphism I50V with recurrent spontaneous abortion (RSA). J Assist Reprod Genet.

[11] Tripathi P, Naik S, Agrawal S (2007). Role of HLA-G, HLA-E and KIR2DL4 in Pregnancy. Int J Hum Genet.

[12] Mallia JV, Das DK, Maitra A (2012). Role of HLA in Human Pregnancy. Int J Hum Genet.

[13] Tang A-W, Alfirevic Z, Quenby S (2011). Natural killer cells and pregnancy outcomes in women with recurrent miscarriage and infertility: a systematic review. Hum Reprod.

[14] Mosaad Y, Abdel‐Dayem Y, El‐Deek B, El‐Sherbini S (2011). Association Between HLA‐E* 0101 Homozygosity and Recurrent Miscarriage in Egyptian Women. Scand J Immunol.

[15] Miller S, Dykes D, Polesky H (1988). A simple salting out procedure for extracting DNA from human nucleated cells. Nucleic Acids Res.

[16] Tripathi P, Naik S, Agrawal S (2006). HLA-E and immunobiology of pregnancy. Tissue Antigens.

[17] Hirankarn N, Kimkong I, Mutirangura A (2004). HLA‐E polymorphism in patients with nasopharyngeal carcinoma. Tissue Antigens.

[18] Strong RK, Holmes MA, Li P, Braun L, Lee N, Geraghty DE (2003). HLA-E allelic variants correlating differential expression, peptide affinities, crystal structures, and thermal stabilities. J Biol Chem.

[19] Gomez-Casado E, Martinez-Laso J, Castro M, Morales P, Trapaga J, Berciano M (1999). Detection of HLA-E and-G DNA alleles for population and disease studies. Cell Mol Life Sci.

[20] Djurisic S, Hviid TV (2014). HLA Class Ib Molecules and Immune Cells in Pregnancy and Preeclampsia. Front Immunol.

[21] Takeshita T (2004). Diagnosis and treatment of recurrent miscarriage associated with immunologic disorders: is paternal lymphocyte immunization a relic of the past?. J Nippon Med School.

[22] Ulbrecht M, Couturier A, Martinozzi S, Pla M, Srivastava R, Peterson PA (1999). Cell surface expression of HLA-E: interaction with human beta2-microglobulin and allelic differences. Eur J Immunol.

[23] Schulte D, Vogel M, Langhans B, Krämer B, Körner C, Nischalke HD (2009). The HLA-ER/HLA-ER Genotype Affects the Natural Course of Hepatitis C Virus (HCV) Infection and Is Associated with HLA-E–Restricted Recognition of an HCV-Derived Peptide by Interferon-γ–Secreting Human CD8+ T Cells. J Infect Dis.

[24] Lajoie J, Hargrove J, Zijenah LS, Humphrey JH, Ward BJ, Roger M (2006). Genetic variants in nonclassical major histocompatibility complex class I human leukocyte antigen (HLA)–E and HLA-G molecules are associated with susceptibility to heterosexual acquisition of HIV-1. J Infect Dis.

[25] Veiga‐Castelli L, Castelli E, Mendes C, da Silva W, Faucher MC, Beauchemin K (2012). Non‐classical HLA‐E gene variability in Brazilians: a nearly invariable locus surrounded by the most variable genes in the human genome. Tissue Antigens.

[26] Maier S, Grzeschik M, Weiss EH, Ulbrecht M (2000). Implications of HLA-E allele expression and different HLA-E ligand diversity for the regulation of NK cells. Hum Immunol.

[27] Grimsley C, Ober C (1997). Population genetic studies of HLA-E: evidence for selection. Hum Immunol.

[28] Matte C, Lacaille J, Zijenah L, Ward B, Roger M, Group ZS (2000). HLA-G and HLA-E polymorphisms in an indigenous African population. Hum Immunol.

[29] Antoun A, Jobson S, Cook M, Moss P, Briggs D (2009). Ethnic variability in human leukocyte antigen‐E haplotypes. Tissue Antigens.

[30] Kimkong I, Mutirangura A, Pimtanothai N (2003). Distribution of human leukocyte antigens-E alleles in Thailand. J Med Assoc Thai.

[31] Steffensen R, Christiansen O, Bennett E, Jersild C (1998). HLA‐E polymorphism in patients with recurrent spontaneous abortion. Tissue Antigens.

[32] Kanai T, Fujii T, Yamashita T, Hyodo H, Miki A, Unno N (2001). Polymorphism of Human Leukocyte Antigen‐E Gene in the Japanese Population with or without Recurrent Abortion. Am J Reprod Immunol.

[33] Hosseini E, Schwarer AP, Jalali A, Ghasemzadeh M (2013). The impact of HLA-E polymorphisms on relapse following allogeneic hematopoietic stem cell transplantation. Leukemia Res.

[34] Hosseini E, Schwarer AP, Ghasemzadeh M (2012). The impact of HLA-E polymorphisms in graft-versus-host disease following HLA-E matched allogeneic hematopoietic stem cell transplantation. Iran J Allergy Asthma Immunol.

